# Supplementary material for: Healthcare Burden of Rare Diseases: A Population-Based Study in Tuscany (Italy)
Source: Int J Environ Res Public Health. 2022 Jun 21;19(13):7553. doi: 10.3390/ijerph19137553 (PMC9265803; doi:10.3390/ijerph19137553)
Supplement: Supplementary file 1 [file ijerph-19-07553-s001.zip › ijerph-1728162-supplementary.pdf]

Table S1. List of rare diseases endowed of an exemption code as defined by the Italian law and included in the study.

INFECTIOUS AND PARASITIC DISEASES	
Disease and/or group	*Specific diseases included in the group*
LEPROSY	
LYME DISEASE	
WHIPPLE DISEASE	
**NEOPLASMS**	
Disease and/or group	*Specific diseases included in the group*
**CARNEY COMPLEX**	
FAMILIAL ADENOMATOUS POLYPOSIS	
GARDNER SYNDROME	
GORLIN SYNDROME RETINOBLASTOMA	
**HEREDITARY NONPOLYPOSIS COLON CANCER**	Lynch syndrome
LYMPHANGIOLEIOMYOMATOSIS	
NEUROFIBROMATOSIS	
NEPHROBLASTOMA	
RETINOBLASTOMA	
**ENDOCRINE DISEASES**	
Disease and/or group	*Specific diseases included in the group*
**ADRENOGENITAL SYNDROME**	
**AUTOIMMUNE POLYENDOCRINOPATHY**	Schmidt's syndrome
CONGENITAL HYPOGONADOTROPIC HYPOGONADISM	
CONGENITAL ISOLATED ACTH DEFICIENCY	
FAMILIAL MEDULLARY THYROID CARCINOMA	
IDIOPATHIC CENTRAL PRECOCIOUS PUBERTY	
KALLMANN SYNDROME	
LARON SYNDROME	
LEPRECHAUNISM	
MULTIPLE ENDOCRINE NEOPLASIA TYPE 1	
MULTIPLE ENDOCRINE NEOPLASIA TYPE 2A	
NON-ACQUIRED ISOLATED GROWTH HORMONE DEFICIENCY	
PENDRED SYNDROME	
REFETOFF SYNDROME	

**RARE PRIMARY HYPERALDOSTERONISM**	Primary hyperaldosteronism-seizures-neurological abnormalities syndrome<br>Primary unilateral adrenal hyperplasia
**METABOLIC DISEASES**	
Disease and/or group	*Specific diseases included in the group*
**DISORDERS OF MITOCHONDRIAL METABOLISM**	
**CREATINE DEFICIENCY SYNDROME**	
KEARNS-SAYRE SYNDROME	
**ISOLATED OXIDATIVE PHOSPHORYLATION COMPLEX DISORDER**	
LEIGH SYNDROME	
LEBER OPTIC ATROPHY	
LONG CHAIN 3-HYDROXYACYL-COA DEHYDROGENASE DEFICIENCY	
MEDIUM CHAIN ACYL-COA DEHYDROGENASE DEFICIENCY	
MELAS	
MERFF	
**MITOCHONDRIAL OXIDATIVE PHOSPHORYLATION DISORDER DUE TO MITOCHONDRIAL DNA ANOMALIES**	
**MITOCHONDRIAL OXIDATIVE PHOSPHORYLATION DISORDER DUE TO NUCLEAR DNA ANOMALIES**	
**OTHER DISORDER OF MITOCHONDRIAL METABOLISM**	Epileptic encephalopathy with global cerebral demyelination
PYRUVATE DEHYDROGENASE PHOSPHATASE DEFICIENCY	
SHORT CHAIN ACYL-COA DEHYDROGENASE DEFICIENCY	
VERY LONG CHAIN ACYL-COA DEHYDROGENASE DEFICIENCY	
**LYSOSOMAL STORAGE DISEASES**	
GALACTOSIALIDOSIS	
**GANGLIOSIDOSIS**	
KRABBE DISEASE	

**LIPID STORAGE DISEASE**	Fabry disease<br>Gaucher disease<br>Niemann-Pick disease<br>Niemann-Pick disease type C
METACHROMATIC LEUKODYSTROPHY	
MUCOLIPIDOSIS	
**MUCOPOLYSACCHARIDOSIS**	Mucopolysaccharidosis type 4
**NEURONAL CEROID LIPOFUSCINOSIS**	
**OTHER LYSOSOMAL STORAGE DISEASES**	Glycogen storage disease due to LAMP-2 deficiency
**DISORDERS OF VITAMIN AND NON-PROTEIN COFACTOR ABSORPTION AND TRANSPORT**	
BIOTINIDASE DEFICIENCY	
**DISORDER OF COBALAMIN AND FOLATE METABOLISM AND TRANSPORT**	Methylmalonic acidemia with homocystinuria, type cblC
**DISORDER OF OTHER VITAMINS AND COFACTORS METABOLISM AND TRANSPORT***<br>*excludes Ataxia with vitamin E deficiency	
HYPOCALCEMIC VITAMIN D-DEPENDENT RICKETS	
HYPOPHOSPHATEMIC RICKETS	
**DISORDERS OF METAL METABOLISM AND TRANSPORT**	
ACERULOPLASMINEMIA	
**DISORDER OF IRON METABOLISM AND TRANSPORT**	Rare hereditary hemochromatosis<br>Hereditary hyperferritinemia-cataract syndrome
MENKES DISEASE	
WILSON DISEASE	
**DISORDERS OF PROTEIN METABOLISM AND TRANSPORT**	
**CONGENITAL DISORDER OF GLYCOSYLATION**	
CRIGLER-NAJJAR SYNDROME	
**PRIMARY SYSTEMIC AMYLOIDOSIS**	Wild type ATTR amyloidosis
**OTHER METABOLIC DISEASES**	
ADIPOSIS DOLOROSA	
ADRENOLEUKODYSTROPHY	

CEREBROTENDINOUS XANTHOMATOSIS	
**DISORDER OF AMINO ACID ABSORPTION AND TRANSPORT**	Albinism<br>Alkaptonuria<br>Cystinosis<br>Cystinuria<br>Glutaric aciduria<br>Glycine encephalopathy<br>Hyperprolinemia<br>Homocystinuria<br>Maple syrup urine disease<br>Methylmalonic acidemia<br>Phenylketonuria/ Hyperphenylalaninemia due to tetrahydrobiopterin deficiency<br>Pyruvate dehydrogenase E1-alpha deficiency<br>Tyrosinemia<br>Other acidemias or primitive organic acidurias from metabolism defects of branched chain amino acids
**DISORDER OF CARBOHYDRATE METABOLISM***<br>*excludes Diabetes mellitus	Fructose-1,6-bisphosphatase deficiency<br>Glucose transport disorder<br>Galactosemia<br>Glycogen storage disease<br>Glycogen storage disease due to acid maltase deficiency<br>Hereditary fructose intolerance
**DISORDER OF LIPID METABOLISM***<br>*excludes Combined familial hyperlipidemia, Dysbetalipoproteinemia, Heterozigous familial hypercholesterolemia type IIA and IIB, Polygenic hypercholesterolemia	Familial lipoprotein lipase deficiency<br>Homozygous familial hypercholesterolemia<br>Familial chylomicronemia syndrome<br>Hypobetalipoproteinemia<br>Tangier disease
DISORDER OF NEUROTRANSMITTER AND PEPTIDE METABOLISM	
**DISORDER OF PURINE OR PYRIMIDINE METABOLISM**	Lesch-Nyhan syndrome
**DISORDER OF UREA CYCLE METABOLISM AND AMMONIA DETOXIFICATION**	Citrullinemia<br>Ornithine transcarbamylase deficiency
FAMILIAL TUMORAL CALCINOSIS	
**FAMILIAL HYPERINSULINISM**	
GENERALIZED LIPODYSTROPHY	
HYPOPHOSPHATASIA	

**PEROXISOMAL DISEASE**	
PORPHYRIA	
REFSUM DISEASE	
ZELLWEGER SYNDROME	
**IMMUNE SYSTEM DISORDERS**	
Disease and/or group	*Specific diseases included in the group*
ACQUIRED ANGIOEDEMA WITH C1INH DEFICIENCY	
ALPHA-1-ANTITRYPSIN DEFICIENCY	
**AUTOINFLAMMATORY SYNDROME**	Periodic fever syndrome<br>Hyperimmunoglobulinemia D with periodic fever<br>Cryopyrin-associated periodic syndrome
**CHRONIC HISTIOCYTOSIS**	Erdheim-Chester disease<br>Langerhans cell histiocytosis<br>Histiocytosis X
FAMILIAL MEDITERRANEAN FEVER	
HEREDITARY ANGIOEDEMA	
NEONATAL ANTIPHOSPHOLIPID SYNDROME	
**PRIMARY IMMUNODEFICIENCY**	Agammaglobulinemia<br>DiGeorge syndrome
SCHNITZLER SYNDROME	
TUMOR NECROSIS FACTOR RECEPTOR 1 ASSOCIATED PERIODIC SYNDROME	
**DISEASES OF THE BLOOD AND BLOOD-FORMING ORGANS**	
Disease and/or group	*Specific diseases included in the group*
CYCLIC NEUTROPENIA	
CHRONIC GRANULOMATOUS DISEASE	
**CHRONIC PRIMARY PLATELET DISORDER**	Immune thrombocytopenia
HEMOLYTIC UREMIC SYNDROME	
**HEREDITARY ANEMIA***<br>*excludes Class I glucose-6-phosphate dehydrogenase deficiency	Blackfan-Diamond anemia<br>Fanconi anemia<br>Hereditary spherocytosis<br>Sickle cell anemia<br>Sideroblastic anemia<br>Thalassemia

**INHERITED COAGULATION DISORDER**	Hemophilia A<br>Hemophilia B<br>Rare hemorrhagic disorder due to a constitutional coagulation factors defect<br>Rare hereditary thrombophilia<br>Von Willebrand disease
**MYELODYSPLASTIC SYNDROME**	Refractory anemia
PAROXYSMAL NOCTURNAL HEMOGLOBINURIA	
RARE ACQUIRED APLASTIC ANEMIA*<br>*excludes Transitory medullary aplasia	
**RARE HEMORRHAGIC DISORDER DUE TO A CONSTITUTIONAL PLATELET ANOMALY**	
**RARE HEMORRHAGIC DISORDER DUE TO A CONSTITUTIONAL THROMBOCYTOPENIA**	
**SEVERE CONGENITAL NEUTROPENIA**	Adult idiopathic neutropenia
SHWACHMAN-DIAMOND SYNDROME	
SYSTEMIC MASTOCYTOSIS	
**PERIPHERAL AND CENTRAL NERVOUS SYSTEM DISORDERS**	
Disease and/or group	*Specific diseases included in the group*
AMYOTROPHIC LATERAL SCLEROSIS	
**AUTOSOMAL DOMINANT CEREBELLAR ATAXIA**	Ataxia with vitamin E deficiency<br>Cerebellar ataxia-hypogonadism syndrome<br>Fragile X-associated tremor/ataxia syndrome<br>Friedreich ataxia<br>Hereditary episodic ataxia<br>Hereditary spastic paraplegia<br>Marie's cerebellar ataxia<br>Marinesco-Sjögren syndrome<br>Pantothenate kinase-associated neurodegeneration
BILATERAL STRIOPALLIDODENTATE CALCINOSIS	
CEREBRAL AUTOSOMAL DOMINANT ARTERIOPATHY-SUBCORTICAL INFARCTS-LEUKOENCEPHALOPATHY	
CHRONIC INFLAMMATORY DEMYELINATING POLYNEUROPATHY	
**CONGENITAL MYASTHENIC SYNDROME**	Myasthenia gravis
**CONGENITAL MYOPATHY**	Central core disease<br>Centronuclear myopathy

	Nemaline myopathy<br>Qualitative or quantitative defects of desmin
DENTATORUBRAL PALLIDOLUYSIAN ATROPHY	
DRAVET SYNDROME	
FAMILIAL OR SPORADIC HEMIPLEGIC MIGRAINE	
**GENETIC PERIPHERAL NEUROPATHY**	Charcot-Marie-Tooth disease/Hereditary motor and sensory neuropathy<br>Dejerine-Sottas syndrome<br>Giant axonal neuropathy<br>Hereditary neuropathy with liability to pressure palsies<br>Hereditary sensory and autonomic neuropathy<br>Roussy-Lévy syndrome
GUILLAIN-BARRÉ SYNDROME	
HUNTINGTON DISEASE	
IDIOPATHIC TORSION DYSTHONIA	
ISAAC SYNDROME	
LAMBERT-EATON MYASTHENIC SYNDROME	
LANDAU-KLEFFNER SYNDROME	
LENNOX-GASTAUT SYNDROME	
**LEUKODYSTROPHY**	Aicardi-Goutières syndrome<br>Alexander disease<br>Pelizaeus-Merzbacher disease
MELKERSSON-ROSENTHAL SYNDROME	
MULTIFOCAL MOTOR NEUROPATHY	
MULTIPLE SYSTEM ATROPHY	
**MUSCULAR DYSTROPHY**	Becker muscular dystrophy<br>Calpain-3-related limb-girdle muscular dystrophy R1<br>Duchenne muscular dystrophy<br>Facioscapulohumeral dystrophy<br>Oculogastrointestinal muscular dystrophy
**MYOTONIC DYSTROPHY**	Paramyotonia congenita of Von Eulenburg<br>Steinert myotonic dystrophy<br>Thomsen and Becker disease
NARCOLEPSY	
**NEURODEGENERATION WITH BRAIN IRON ACCUMULATION**	Infantile neuroaxonal dystrophy

NEURONAL INTRANUCLEAR INCLUSION DISEASE	
**PERIODIC PARALYSIS**	
POEMS SYNDROME	
**PRIMARY DYSTONIA**	
PRIMARY LATERAL SCLEROSIS	
PROGRESSIVE MYOCLONIC EPILEPSY	
PROGRESSIVE SUPRANUCLEAR PALSY	
RETT SYNDROME	
RIBOFLAVIN TRANSPORTER DEFICIENCY	
**SPINAL MUSCULAR ATROPHY**	Kennedy disease<br>Proximal spinal muscular atrophy type 1<br>Proximal spinal muscular atrophy type 3
STIFF PERSON SPECTRUM DISORDER	
SYRINGOMYELIA	
WEST SYNDROME	
**DISORDERS OF THE EYE AND ADNEXA**	
Disease and/or group	*Specific diseases included in the group*
COGAN SYNDROME	
CONGENITAL STATIONARY NIGHT BLINDNESS	
**CORNEAL DYSTROPHY**	Epithelial basement membrane dystrophy<br>Fuchs endothelial corneal dystrophy<br>Granular corneal dystrophy type II<br>Macular corneal dystrophy<br>Meesmann corneal dystrophy<br>Posterior polymorphous corneal dystrophy<br>Reis-Bücklers corneal dystrophy
FAMILIAL EXUDATIVE VITREORETINOPATHY	
**ISOLATED CHORIORETINAL DYSTROPHY**	
**ISOLATED INHERITED RETINAL DISORDER**	Best vitelliform macular dystrophy<br>Leber congenital amaurosis<br>Progressive cone dystrophy<br>Retinitis pigmentosa<br>Retinitis punctata albescens<br>Stargardt disease<br>Vitreoretinal distrophy

OGUCHI DISEASE	
POSTERIOR UVEITIS	
SERPIGINOUS CHOROIDITIS	
SYNDROMIC KERATOCONUS	
**CIRCULATORY SYSTEM DISEASES**	
Disease and/or group	*Specific diseases included in the group*
ANTI-GLOMERULAR BASEMENT MEMBRANE DISEASE	
BEHÇET DISEASE	
BUDD-CHIARI SYNDROME	
CRYOGLOBULINEMIC VASCULITIS	
EOSINOPHILIC GRANULOMATOSIS WITH POLYANGIITIS	
GIANT CELL ARTERITIS	
GRANULOMATOSIS WITH POLYANGIITIS	
HEREDITARY HEMORRHAGIC TELANGIECTASIA	
IMMUNOGLOBULIN A VASCULITIS	
MICROSCOPIC POLYANGIITIS	
POLYARTERITIS NODOSA	
**PRIMARY LYMPHEDEMA**	Milroy disease<br>Meige disease<br>Idiopathic primary lymphedema<br>Recessive primary congenital lymphedema
RHEUMATIC HEART DISEASE	
TAKAYASU ARTERITIS	
**THROMBOTIC MICROANGIOPATHY**	Hemolytic uremic syndrome<br>Thrombotic thrombocytopenic purpura
**RESPIRATORY DISEASES**	
Disease and/or group	*Specific diseases included in the group*
AUTOIMMUNE PULMONARY ALVEOLAR PROTEINOSIS	
IDIOPATHIC PULMONARY ARTERIAL HYPERTENSION	
IDIOPATHIC PULMONARY HEMOSIDEROSIS	
ONDINE SYNDROME	
PRIMARY CILIARY DYSKINESIA	
PRIMARY CILIARY DYSKINESIA, KARTAGENER TYPE	

**PRIMARY INTERSTITIAL LUNG DISEASE IN CHILDHOOD AND ADULTHOOD**	Acute interstitial pneumonia<br>Idiopathic pulmonary fibrosis
SARCOIDOSIS	
**DIGESTIVE DISORDERS**	
Disease and/or group	*Specific diseases included in the group*
CHRONIC INTESTINAL PSEUDOOBSTRUCTION	
CONGENITAL CHLORIDE DIARRHEA	
EOSINOPHILIC GASTROENTERITIS	
**ISOLATED ACHALASIA AND SYNDROMES-ASSOCIATED ACHALASIA**	Triple A syndrome
PRIMARY INTESTINAL LYMPHANGIECTASIA	
PRIMARY SCLEROSING CHOLANGITIS	
**PROGRESSIVE FAMILIAL INTRAHEPATIC CHOLESTASIS**	Progressive familial intrahepatic cholestasis type 3
**DISEASES OF THE GENITOURINARY SYSTEM**	
Disease and/or group	*Specific diseases included in the group*
ALPORT SYNDROME	
IGG4-RELATED RETROPERITONEAL FIBROSIS	
INTERSTITIAL CYSTITIS	
NEPHROGENIC DIABETES INSIPIDUS	
**PRIMARY GLOMERULAR DISEASE**	
**RARE RENAL TUBULAR DISEASE**	Bartter syndrome<br>Dent disease<br>Gitelman syndrome
**DISEASES OF THE SKIN AND SUBCUTANEOUS TISSUE**	
Disease and/or group	*Specific diseases included in the group*
APLASIA CUTIS CONGENITA	
BULLOUS PEMPHIGOID	
CONGENITAL NON-BULLOUS ICHTHYOSIFORM ERYTHRODERMA	
CUTIS LAXA	
DARIER DISEASE	
DIFFUSE CUTANEOUS SYSTEMIC SCLEROSIS	

DYSKERATOSIS CONGENITA	
EEC SYNDROME	
**ECTODERMAL DYSPLASIA SYNDROME**	
EPIDERMAL NEVUS SYNDROME	
ERYTHROKERATODERMIA VARIABILIS	
FAMILIAL ATYPICAL MULTIPLE MOLE MELANOMA SYNDROME	
**HEREDITARY PALMOPLANTAR KERATODERMA**	
HYPOMELANOSIS OF ITO	
IBIDS SYNDROME	
INCONTINENTIA PIGMENTI	
INHERITED EPIDERMOLYSIS BULLOSA	
**INHERITED ICHTHYOSIS**	Autosomal recessive congenital ichthyosis<br>Lamellar ichthyosis<br>Netherton syndrome<br>X-linked ichthyosis
KERATOSIS FOLLICULARIS SPINULOSA DECALVANS	
LICHEN SCLEROSUS ET ATROPHICUS	
MUCOUS MEMBRANE PEMPHIGOID	
PEMPHIGUS	
PSEUDOXANTHOMA ELASTICUM	
PYODERMA GANGRENOSUM	
SJÖGREN-LARSSON SYNDROME	

DISEASES OF THE MUSCULOSKELETAL SYSTEM AND CONNECTIVE TISSUE

Disease and/or group	*Specific diseases included in the group*
ANTISYNTHETASE SYNDROME	
DERMATOMYOSITIS	
DIFFUSE FASCIITIS	
EOSINOPHILIC FASCIITIS	
FIBRODYSPLASIA OSSIFICANS PROGRESSIVA	
GORHAM-STOUT DISEASE	
INCLUSION BODY MYOSITIS	
MIXED CONNECTIVE TISSUE DISEASE	
POLYMYOSITIS	
RELAPSING POLYCHONDRITIS	
SAPHO SYNDROME	

SYSTEMIC SCLEROSIS	
**CONGENITAL ANOMALIES, CHROMOSOMAL ABERRATIONS AND GENETIC SYNDROMES**	
Disease and/or group	*Specific diseases included in the group*
**CONGENITAL MALFORMATIONS OF THE NERVOUS SYSTEM**	
ARNOLD-CHIARI MALFORMATION	
CEREBELLAR AGENESIS	
GERSTMANN SYNDROME	
ISOLATED OR SYNDROMIC HOLOPROSENCEPHALY	
ISOLATED OR SYNDROMIC MICROCEPHALY	
JOUBERT SYNDROME AND RELATED DISORDERS	
LISSENCEPHALY	
**SYNDROME WITH CORPUS CALLOSUM AGENESIS/DYSGENESIS AS A MAJOR FEATURE**	Dandy-Walker sydrome
WALKER-WARBURG SYNDROME	
**OTHER SEVERE AND DISABLING SYNDROMES WITH A CENTRAL NERVOUS SYSTEM MALFORMATION AS A MAJOR FEATURE**	
**CONGENITAL MALFORMATIONS OF THE EYE**	
ANIRIDIA	
AXENFELD-RIEGER SYNDROME	
COLOBOMA OF THE OPTIC NERVE	
**ISOLATED OR SYNDROMIC CONGENITAL OCULAR COLOBOMA**	
**MICROPHTHALMIA-ANOPHTHALMIA-COLOBOMA**	Microphthalmia, Lenz type
PETERS ANOMALY	
RIEGER ANOMALY	
SEPTO-OPTIC DYSPLASIA SPECTRUM	
VOGT-KOYANAGI-HARADA DISEASE	
**ISOLATED OR SYNDROMIC CONGENITAL CRANIOFACIAL ANOMALIES**	
ACROCEPHALOSYNDACTYLY	
ANTLEY-BIXLER SYNDROME	
APERT SYNDROME	

PFEIFFER SYNDROME	
PIERRE ROBIN SYNDROME	
**OTHER SEVERE AND DISABLING ANOMALIES OF SKULL AND FACE BONES, INTEGUMENTS AND MUCOSA**	Crouzon disease<br>Frontofacionasal dysplasia<br>Isolated craniosynostosis<br>Isolated or syndromic cleft palate
**CONGENITAL FACIAL ABNORMALITIES**	
GOLDENHAR SYNDROME	
MOEBIUS SYNDROME	
SCHINZEL-GIEDION SYNDROME	
**CONGENITAL LIMB MALFORMATIONS**	
ADAMS-OLIVER SYNDROME	
**ARTHROGRYPOSIS MULTIPLEX CONGENITA**	
CONGENITAL ABSENCE OF UPPER ARM AND FOREARM WITH HAND PRESENT	
FEMORAL-FACIAL SYNDROME	
FREEMAN-SHELDON SYNDROME	
POLAND SYNDROME	
**CONGENITAL HEART DISEASE**	
KLIPPEL-TRÉNAUNAY SYNDROME	
**RARE CONGENITAL NON-SYNDROMIC HEART MALFORMATION***<br>*excludes Isolated atrial septal defect, Isolated pulmonary valve stenosis, Isolated ventricular septal defect, Patent ductus arteriosus	Ebstein malformation of the tricuspid valve<br>Hypoplastic left heart syndrome
**OTHER SEVERE AND DISABLING CONGENITAL SYNDROMIC VASCULAR DISEASE**	CLOVES syndrome<br>Capillary malformation-arteriovenous malformation
**ABDOMINAL WALL DEFECTS**	
ISOLATED KLIPPEL-FEIL SYNDROME	
**CONGENITAL ANOMALIES OF THE GASTROINTESTINAL TRACT**	
ANORECTAL MALFORMATION	
CAROLI DISEASE	
DUPLICATION OF THE GASTROINTESTINAL TRACT	

ESOPHAGEAL ATRESIA AND/OR ISOLATED TRACHEOESOPHAGEAL FISTULA	
ISOLATED BILIARY ATRESIA	
ISOLATED POLYCYSTIC LIVER DISEASE	
HIRSCHSPRUNG DISEASE	
PERSISTENT CLOACA	
**CONGENITAL UROGENITAL ANOMALIES**	
AUTOSOMAL RECESSIVE POLYCYSTIC KIDNEY DISEASE	
BLADDER EXSTROPHY	
**DISORDER OF SEXUAL DEVELOPMENT AND/OR AMBIGUOUS GENITALIA AND/OR KARIOTYPE DISCORDANCE/GONADAL DEVELOPMENT AND/OR PHENOTYPE**	Gonadal dysgenesis<br>Complete androgen insensitivity syndrome<br>Partial androgen insensitivity syndrome
MAYER-ROKITANSKY-KÜSTER-HAUSER SYNDROME	
**FAMILIAL CYSTIC RENAL DISEASE**	
MEDULLARY SPONGE KIDNEY	
**PSEUDOHERMAPHRODITISM**	
**OTHER SEVERE AND DISABLING DEFECTS OF SEXUAL DEVELOPMENT AND/OR AMBIGUOUS GENITALIA AND/OR KARIOTYPE DISCORDANCE/GONADAL DEVELOPMENT AND/OR PHENOTYPE**	
**GENETIC SKELETAL DISORDERS**	
ACRODYSOSTOSIS	
**CONGENITAL CHONDRODISPLASIAS**	Achondroplasia<br>Jeune syndrome<br>Multiple osteochondromas
**CONGENITAL ISOLATED OR SYNDROMIC CHONDRODISPLASIAS**	Camurati-Engelmann disease<br>Craniometaphyseal dysplasia<br>Fibrous dysplasia<br>Léri-Weill dyschondrosteosis<br>Multiple epiphyseal dysplasia<br>McCune-Albright syndrome<br>Osteogenesis imperfecta<br>Osteopetrosis and related disorders<br>Spondyloepiphyseal dysplasia tarda
MAFFUCCI SYNDROME	

SPONDYLOEPIPHYSEAL DYSPLASIA CONGENITA	
**OTHER SYNDROMES AND COMPLEX CONGENITAL MALFORMATIONS**	
22q11.2 DELETION SYNDROME	
AARSKOG-SCOTT SYNDROME	
ALAGILLE SYNDROME	
ALSTRÖM SYNDROME	
ANGELMAN SYNDROME	
BARDET-BIEDL SYNDROME	
BECKWITH-WIEDEMANN SYNDROME	
BOR SYNDROME	
BORJESON-FORSSMAN-LEHMANN SYNDROME	
BRANCHIO-OCULO-FACIAL SYNDROME	
CARDIOFACIOCUTANEOUS SYNDROME	
CHAR SYNDROME	
CHARGE SYNDROME	
COCKAYNE SYNDROME	
COFFIN-SIRIS SYNDROME	
COHEN SYNDROME	
CORNELIA DE LANGE SYNDROME	
DUBOWITZ SYNDROME	
EHLERS-DANLOS SYNDROME	
FRAGILE X SYNDROME	
HOLT-ORAM SYNDROME	
ISOLATED HEMIHYPERPLASIA	
KABUKI SYNDROME	
LOEYS-DIETZ SYNDROME	
MARFAN SYNDROME	
MARSHALL SYNDROME	
MARSHALL-SMITH SYNDROME	
MONOSOMY 5P	
**MOSAIC VARIEGATED ANEUPLOIDY SYNDROME**	
**MULTIPLE CONGENITAL ANOMALIES/DYSMORPHIC SYNDROME-INTELLECTUAL DISABILITY**	KBG syndrome
**MULTIPLE HAMARTOMAS**	Cowden syndrome<br>Hepatic cystic hamartoma

NAIL-PATELLA SYNDROME	
NOONAN SYNDROME AND NOONAN-RELATED SYNDROME	
NOONAN SYNDROME WITH MULTIPLE LENTIGINES	
OCULODENTODIGITAL DYSPLASIA	
OPITZ GBBB SYNDROME	
PEUTZ-JEGHERS SYNDROME	
PRADER-WILLI SYNDROME	
PROGRESSIVE HEMIFACIAL ATROPHY	
RUBINSTEIN-TAYBI SYNDROME	
SALDINO-MAINZER SYNDROME	
SECKEL SYNDROME	
SILVER-RUSSELL SYNDROME	
SMITH-MAGENIS SYNDROME	
SOTOS SYNDROME	
STICKLER SYNDROME	
STURGE-WEBER SYNDROME	
**SYNDROMIC CHROMOSOMAL AND GENOMIC UNBALANCED REARRANGEMENTS**	Distal 22q11.2 microdeletion syndrome
TETRASOMY 12P	
TOWNES-BROCKS SYNDROME	
TUBEROUS SCLEROSIS COMPLEX	
TURNER SYNDROME	
VACTERL/VATER ASSOCIATION	
VON HIPPEL-LINDAU DISEASE	
WAGR SYNDROME	
**WAARDENBURG SYNDROME**	
WILLIAMS SYNDROME	
WOLF-HIRSCHHORN SYNDROME	
WOLFRAM SYNDROME	

NEONATAL MORBITIES OF PERINATAL ORIGIN

Disease and/or group	*Specific diseases included in the group*
BILIRUBIN ENCEPHALOPATHY	
FETAL ALCOHOL SYNDROME	
HEPATIC FIBROSIS-RENAL CYSTS-INTELLECTUAL DISABILITY SYNDROME	

For each nosological group, groups of diseases are reported in bold and, if available, specific diseases belonging to the groups are indicated.
